# A Highly Stable and Sustainable Low-Temperature Selective Absorber: Structural and Ageing Characterisation

**DOI:** 10.3390/ma15103427

**Published:** 2022-05-10

**Authors:** Meryem Farchado, Gema San Vicente, Nuria Germán, César Maffiotte, Ángel Morales

**Affiliations:** 1 Unidad de Materiales para Tecnologías Termosolares de Concentración, Plataforma Solar de Almería, Departamento de Energía, Centro de Investigaciones Energéticas, Medioambientales y Tecnológicas (CIEMAT), Avda. Complutense 40, 28040 Madrid, Spain; gema.sanvicente@ciemat.es (G.S.V.); n.german@ciemat.es (N.G.); angel.morales@ciemat.es (Á.M.); 2 División de Materiales de Interés Energético, Departamento de Tecnología, Centro de Investigaciones Energéticas, Medioambientales y Tecnológicas (CIEMAT), Avda. Complutense 40, 28040 Madrid, Spain; cesar.maffiotte@ciemat.es

**Keywords:** solar selective absorber, spinel, dip-coating, structural properties, durability

## Abstract

Solar absorbers in a three-layer configuration have been prepared by dip-coating onto aluminium substrates. They are constituted by two spinel layers with one silica layer on the top and values of solar absorptance above 0.950 and thermal emittance below 0.04 were obtained. The effects of using different sintering conditions of the upper silica layer on the optical behaviour and durability tests have been studied. Results obtained in accelerated ageing methods, such as thermal stability tests and condensation tests, clearly show that the proposed selective absorber exhibits excellent thermal stability and very good humidity resistance. The results show that the protective action is due not only to the silica layer but also to the alumina layer produced during the absorber preparation. The phase composition of the individual layers was independently confirmed using X-ray diffraction and corroborated by X-ray Photoelectron Spectroscopy. Spinel-like phases were obtained in both the first and second layers. The ageing study shows that the three-layer configuration proposed has a very high potential, in terms of both durability and optical behaviour, for solar thermal low-temperature applications.

## 1. Introduction

Energy demands and global warming are increasing as a consequence of the use of conventional energy sources (fossil fuel, gasoline, gas and charcoal) that satisfy around 80% of the global demand for energy [1]. During the last few years, the total carbon dioxide emissions have increased by 54% demonstrating the dominance of fossil fuels worldwide [2,3]. Given the current energy crisis and the critical environmental concerns as exposed in the Paris Agreement in 2015, it is indispensable to promote the development of sustainable sources of energy for reducing polluting emissions, increasing renewable electricity production and improving energy efficiency. This decarbonisation of the economy would be achieved with the use of renewable energies. Solar thermal energy, which uses the full-solar spectrum without greenhouse gas emissions for producing heat, can be a potential candidate to face this universal issue by replacing fossil fuels. 

Focusing on low and medium temperature solar applications, several new designs have been developed during the last three decades in order to enhance the thermal performance of flat plate collectors, which are widely used in water and space heating [4]. The success of this technology relies on the solar absorber material that has to combine high optical efficiency with long-term performance stability. The optical requirement that the material must accomplish is a high selectivity, which is achieved when a high solar absorptance (α_s_) in the solar wavelength range (0.3–2.5 μm), and a low thermal emittance (ε_T_) in the mid/far-infrared wavelength ranges (>2.5 μm) is realised [5]. Solar selective coatings, specifically those based on spinel-type mixed oxides, are widely used in the field of solar thermal energy conversion in order to ensure a greater output of the collector. Apart from the well-known field of selective absorbers, spinel-type mixed oxides have been reported to be suitable for several catalytic processes, thermochemical energy storage and protective coating applications, among others [6,7,8]. This growing interest in spinel-type transition metal oxides in many research fields is due to their broad variety of physical and chemical properties. 

Spinel structures have the possibility of substituting a large number of transition metals and of replacing the cations present in the spinel crystal lattice, in both tetrahedral and octahedral sites, which allows chemical modifications and therefore the tuning of their properties [9,10]. This can be achieved as the physicochemical properties of the spinel-structure compounds rigorously depend on the type, charge, and distribution in tetrahedral/octahedral sites of the cations [11,12]. This remarkable peculiarity in terms of the huge versatility of the spinel coatings as well as their inherent high resistance to oxidation and high temperature stability has generated special interest in the field of spectrally selective absorber coatings during the last decades [13,14,15]. 

This current interest is reflected in our previous published work in which a multi-layered absorber for low-temperature thermal applications based on CuMnO_x_ and two antireflective coatings deposited by the dip-coating method on an etched aluminium substrate (Al-CuMnO_x_/CuFeMnO_x_/SiO_2_) was reported [16]. The good spectral selectivity achieved, with solar absorptance of 0.957 and thermal emittance at 100 °C of 0.038, demonstrated the possibility of this three-layer configuration selective absorber to be considered as a future competitive material by the industry. However, before further development or commercialisation, it is critical to evaluate the durability of the solar absorber in terms of performance during the collector’s lifetime. The service life of a material depends on several variables which influence its behaviour under service conditions such as its physical and chemical properties, its performance requirement in the application considered, and on external environmental factors. Consequently, apart from high spectral selectivity (high absorptance and low emittance in the range of 0.3–2.5 μm and 2.5–17 μm, respectively), solar selective coatings have to possess high chemical and thermal stabilities at typical field operation temperatures or under regular variations in operating temperature [17]. 

In the case of low-temperature solar collectors, as they operate in outdoor conditions, it is indispensable for these systems to withstand external environmental factors such as high humidity, high temperatures, atmospheric corrosion and ultraviolet irradiance or wind and snow loads depending on the geographic position [18,19,20]. Focusing on the selective absorber coatings composed of inorganic oxides, high temperatures can speed up oxidation processes and high levels of humidity may create hydrolytic reactions (electrochemical corrosion) further oxidating the surface [21,22,23,24,25]. It should be taken into account that each of these manifold micro-climatic influences can degrade the absorber and the rest of the components of a collector, causing the absorber’s optical properties to deteriorate and the loss of the absorber performance over the years [26,27].

In this work, the influence of the SiO_2_ layer sintering on the optical parameters and long-term durability of the proposed Al/CuMnO_x_/CuFeMnO_x_/SiO_2_ selective absorber is reported. The structural analysis of each constituent layer of the selective absorber by X-ray diffraction (XRD) and X-ray photoelectron spectroscopy (XPS) is also included.

## 2. Materials and Methods

### 2.1. Sample Preparation

A chemical etching treatment (10 s in a 5% HF solution) of the aluminium substrate was carried out before depositing the constituent absorber films. Next, each layer of the selective absorber (CuMnO_x_/CuFeMnO_x_/SiO_2_) was separately deposited by dip-coating at 22 °C. All the solutions were kept at this temperature during the absorber preparation to ensure a constant viscosity.

The CuMnO_x_ and CuFeMnO_x_ solutions were obtained by dissolving the metallic precursors Cu(NO_3_)_2_ (Sigma Aldrich, St. Louis, MO, USA, 98–103%), Mn(NO_3_)_2_ (Panreac, 97%) and Fe(NO_3_)_3_ (PRS Panreac, 98%) in absolute ethanol at molar ratios equal to 1:1 for CuMnO_x_ and 2:1:1 for CuFeMnO_x_. In order to stabilise these precursor alcoholic solutions and improve the adhesion of liquid-films, some reagents such as acid, complexing agent and wetting additive were added [28]. Regarding the SiO_2_ anti-reflective coating, the solution was prepared via sol-gel by mixing the precursor Tetraethylortosilicate (TEOS) with distilled water and ethanol in a molar ratio of 1:18:5 [16] with hydrochloric acid added as catalyst. 

The three layers deposition was performed at the optimised withdrawal rates of 42 cm/min for the CuMnO_x_ film, 18 cm/min for the first CuFeMnO_x_ antireflective coating and 12 cm/min for the SiO_2_ anti-reflective coating. These rates were selected as optimal after carefully studying the effect of the withdrawal rate and, therefore, the thickness of each constituent layer in the optical properties (high solar absorptance and low thermal emittance) [16]. The resulting layers of CuMnO_x_ and CuFeMnO_x_ were annealed separately at 600 °C. Regarding the SiO_2_ layer, different sintering conditions (blower or oven, 5 or 15–30 min) were tested at 500 °C in order to study the possible influence of this parameter on the complete solar absorber after long term durability tests. Moreover, the condensation resistance of the SiO_2_ layer sintered at the different conditions was also studied independently by preparing this layer directly on the aluminium substrate.

### 2.2. Sample Characterisation

A Pananlytical XPERT X-ray diffraction spectrometer (XRD) was used for characterising the constituent absorber thin films. The structural analysis was carried out by identifying the crystalline phases composing the coatings. Additionally, a Perkin-Elmer PHI 5400 X-ray photoelectron spectrometer (XPS) equipped with a Mg Kα excitation source (hν = 1253.6 eV) and a beam size of 1 mm diameter was used to determine the composition of the films and to identify the valence state and cation distribution of the components present in each layer. The intensities were estimated by calculating the area under each peak after smoothing and removing the background using the modified method of Shirley and fitting the experimental curve to a Gaussian–Lorentzian ratio variable curve using an iterative algorithm. Depth profiling analysis was obtained by sputtering the complete three-layer absorber with a 4.0 keV Argon ion flux at 1.5 eV acceleration voltage.

The condensation ageing experiment was performed in a Q.U.V. weathering chamber following the procedure developed by ISO 22975-3 (2014) standard [29]. The samples were subjected to condensation conditions at 40 °C for 150 h, 300 h and 600 h in order to determine the resistance to condensed water of the selective absorber. A thermal stability test was also carried out by using a conventional circulating air furnace where the samples were first heated at 250 °C for 200 h and then at 300 °C for 200 h, 400 h, 600 h and 800 h.

The optical properties of the absorbers were measured before and after the ageing studies. Regarding α_s_, this parameter was calculated as specified by the standard procedure [30] by using the direct AM1.5 solar spectrum from ASTM G173-03 in the 0.3–2.5 µm range [31] from solar hemispherical reflectance values (ρ_s,h_). The hemispherical reflectance spectra of the samples were recorded from 0.3 to 2.5 µm with a UV-VIS-NIR Perkin-Elmer LAMBDA 950 double beam spectrophotometer equipped with a 150 mm Spectralon^®^ coated integrating sphere. The associated measurement uncertainty was 1%. 

Samples ε_T_ was also determined as specified by the standard procedure [29]. In this case, a Perkin Elmer Frontier FTIR spectrophotometer equipped with a diffuse gold-coated integrating sphere was used. A certified infragold standard was used as the reference material. The accuracy of the reflectance data was estimated to be 2% for the FTIR-spectrophotometer. Thermal emittance was calculated from the hemispherical IR-reflectance spectra recorded from 2.5 to 17 µm measured at room temperature and the black body spectrum at 100 °C. 

For defining the acceptable durability or service life of the absorber, the performance criterion (PC) was determined. This parameter was calculated according to the ISO 22975-3 (2014) standard [29], that is, from the optical properties before and after the accelerated ageing test by applying Equation (1) in which the importance of the thermal emittance is reduced by a factor of 0.5:

PC = − Δα_s_ + 0.50 Δε_t_ ≤ 0.05 (1)

where Δα_s_ is the change in the solar absorptance defined as Δα_s_= α_s,t_ − α_s,i_ being α_s,t_ the solar absorptance at the actual time of the test or service, and α_s,i_ the initial value of solar absorptance. Δε_t_ is the change in the thermal emittance defined as Δε= ε_t_ − ε_i_ being ε_t_ the thermal emittance at the actual time of the test or service, and ε_i_ the initial value of thermal emittance. 

## 3. Results and Discussion

### 3.1. Structural Study of the Al/CuMnO_x_/CuFeMnO_x_/SiO_2_ Absorber

In order to study the crystalline structure and the composition of each constituent layer of the selective absorber, grazing angle XRD and XPS measurements were performed, respectively.

#### 3.1.1. XRD Analysis

**Figure 1:**
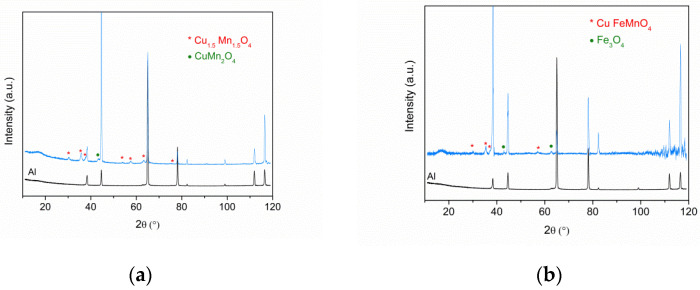
(**a**) XRD spectra of etched aluminium substrate and the CuMnO_x_ film sintered at 600 °C; (**b**) XRD spectra of etched aluminium substrate and the CuFeMnO_x_ coating sintered at 600 °C.

XRD patterns of the complete selective absorber were obtained where various peak characteristics of spinel phases could be identified. However, the interpretation of each layer’s contribution to these spectra is very difficult due to peaks of spinel with different stoichiometry and composition occurring close to each other and the high intensity of the substrate (aluminium) peaks. Therefore, the XRD study of each film of the absorber was carried out separately. Figure 1 shows the XRD spectra obtained for Al/CuMnO_x_ (a) and Al/CuFeMnO_x_ (b) samples.

These spectra show a clear crystallisation for both CuMnO_x_ and CuFeMnO_x_ films as indicated by the presence of characteristic peaks. 

It should be noticed that the signals obtained for the films were less intense than the aluminium substrate ones. This is due to the larger proportion of aluminium substrate in the sample compared to the coatings whose thicknesses were only a few nanometers (70 nm for CuMnO_x_ and 30 nm for CuFeMnO_x_) [16]. Similar results were obtained by Amri et al. [32] who needed up to six dip-heating cycles in order to obtain spinel peaks of similar intensity as the aluminium peaks. For this reason and in order to ensure a clear visualisation of the XRD patterns related to the constituent absorber coatings, the diffractogram of the aluminium substrate is incorporated in each graphical representation. Concerning the peaks of the aluminium substrate, the identification was carried out by using the reference codes ICDD 00-001-1179 and ICDD 03-065-2869.

Based on the XRD pattern of the CuMnO_x_ coating shown in Figure 1a, the first constituent absorber layer is composed of two coexisting crystalline phases: the spinel-type structure of Cu_1_._5_Mn_1_._5_O_4_ (ICDD 01-070-0262) and a secondary CuMn_2_O_4_ spinel phase (ICDD 01-076-2296). Despite both crystalline phases coexisting in the CuMnO_x_ film, the CuMn_2_O_4_ phase is expected to be a minority in this coating compared to the Cu_1_._5_Mn_1_._5_O_4_ spinel-type phase since only one weak diffraction peak at 43° is assigned to the CuMn_2_O_4_ spinel in the diffractogram. All other peaks, (discarding the peaks allocated to the aluminium) are assigned to the Cu_1_._5_Mn_1_._5_O_4_ spinel phase.

In the same manner and in accordance with the ICDD database, the XRD results concerning the CuFeMnO_x_ film indicate a mixture of two crystalline phases. As it is shown in Figure 1b, both coexisting phases correspond to the spinel-type structure of CuFeMnO_4_ (ICDD 00-020-0358) and the magnetite phase Fe_3_O_4_ (ICDD 00-001-1111).

In the case of SiO_2_coating, no crystalline structure was identified in the XRD analysis for any of the different thermal treatments studied, that is, blower for 5 min or oven for 15–30 min. These results were expected due to the soft thermal treatments to which the SiO_2_ anti-reflective coating has been subjected for its sintering stage.

Definitely, XRD spectra confirmed the co-existence of two spinel-type phases in the first constituent absorber layer (Cu_1_._5_Mn_1_._5_O_4_ and CuMn_2_O_4_) and the co-existence of both CuFeMnO_4_ spinel-type and Fe_3_O_4_ magnetite phases in the second constituent absorber layer. Meanwhile, the SiO_2_ over-coating was found to be amorphous. In no case peaks corresponding to alumina appeared, indicating that this layer formed during the sintering of each constituent layer was amorphous.

#### 3.1.2. XPS Analysis

In order to confirm and complete the results obtained by XRD, an additional study by XPS was carried out on the CuMnO_x_, CuFeMnO_x_ and SiO_2_ coatings. This study allowed us to determine the element-binding states and the composition of the three constituent absorber layers.

**Figure 2:**
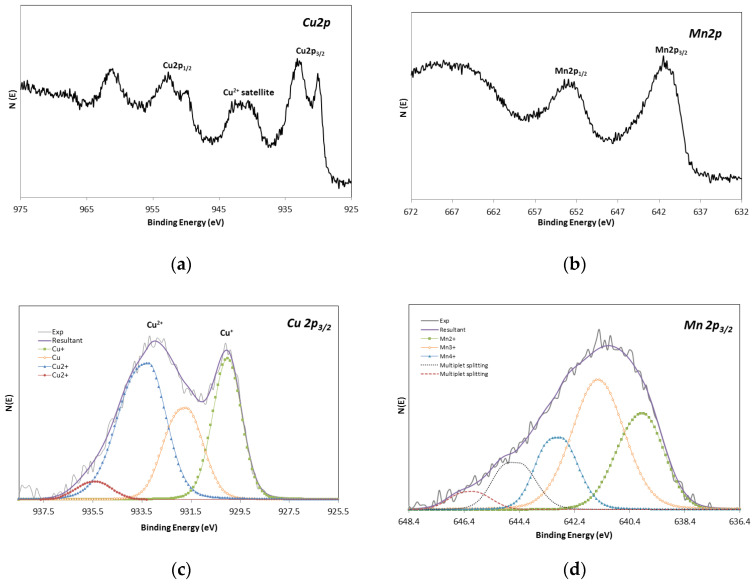
XPS spectra of Cu 2p (**a**), Mn 2p (**b**) and the congruent fit curves of Cu 2p_3/2_ (**c**) and Mn 2p_3/2_ (**d**) peaks for CuMnO_x_ film annealed at 600 °C.

**Figure 3:**
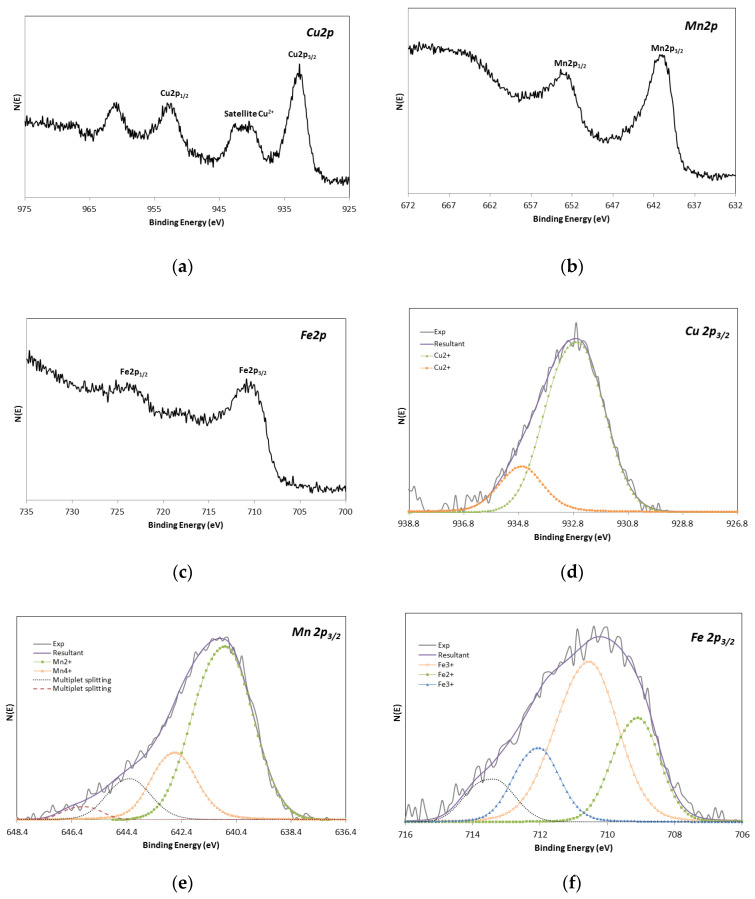
XPS spectra of Cu 2p (**a**), Mn 2p (**b**), Fe 2p (**c**) and the corresponding fit curves of Cu 2p_3/2_ (**d**), Mn 2p_3/2_ (**e**) and Fe 2p_3/2_ (**f**) peaks for CuFeMnO_x_ film annealed at 600 °C. In (**e**,**f**) the percentages of Mn and Fe species were recalculated once the multiplet split components (higher than 644 eV (Mn)) and satellite contributions (at 713.4 eV (Fe)) were removed.

- CuMnO_x_
  For the CuMnO_x_ deposited layer, a metallic atomic ratio of Cu (16.6%)/Mn (16.8%) = 0.99 was obtained in the XPS analysis. These values are in accordance with the selected metallic ratio in the precursor solution (Cu:Mn 1:1). Additionally, the XPS technique also provided information about the oxidation state of Cu and Mn ions by studying their corresponding 2p_3/2_ core level peak. Figure 2 shows the XPS spectra of Cu 2p (a), Mn 2p (b) and the congruent fit curves of Cu 2p_3/2_ (c) and Mn 2p_3/2_ (d) peaks recorded for the CuMnO_x_ film annealed at 600 °C. As can be seen, the peaks associated with the metals present in the film are significantly complex. Therefore, contributions of various oxidation states are expected at first sight.
  Based on the Cu ion contribution in the CuMnO_x_ film, four differentiated peaks in the deconvolution curve of Cu 2p_3/2_ are observed. The peak at the low binding energy of 930.0 eV can be assigned to the Cu^+^ ion in the Cu_1_._5_Mn_1_._5_O_4_ spinel structure [33,34]. A second peak at 931.8 eV could be assigned to metallic Cu [35] taking into account its Cu LMM kinetic energy (918.0 eV, spectrum not shown here). Finally, two more peaks at 933.3 and 935.5 eV can be referred to the Cu^2+^ spectrum [33,36]. The large shake-up satellite peak registered between 938 and 945 eV in the Cu2p spectrum (Figure 2a) confirms the presence of Cu^2+^. The identification of peaks related to the same Cu^2+^ species but at different positions is attributable to the existence of a different chemical environment of this ion in the spinel crystal lattice since these ions can occupy both octahedral and tetrahedral sites [10]. Specifically, these different contributions of the Cu^2+^ ion in the CuMnO_x_ film support the results obtained in the XRD analysis in which two types of spinel phases were detected: Cu_1_._5_Mn_1_._5_O_4_ and CuMn_2_O_4_. In relation to the site distribution of Cu ions, it can be assigned in accordance with the majority of the references that Cu^+^ occupies tetrahedral sites (935.0 eV) while the Cu^2+^ occupies preferably octahedral sites (933.3 eV) [11,34,37,38]. Additionally, by analysing the quantified results shown in Figure 2c, it can be seen that the Cu^2+^ dominates in the CuMnO_x_ film against the Cu^+^ species.
  Regarding the second constituent species of the CuMnO_x_ film, it is observed that the deconvolution curve of Mn 2p_3/2_ is composed of three peaks at binding energies of 640.0, 641.5 and 642.9 eV corresponding to Mn^2+^, Mn^3+^ and Mn^4+^ [17,38,39,40], respectively, and multiplet split components associated with Mn oxides at binding energies higher than 644 eV [17,38,39]. Regarding the site distribution of manganese ions, the majority of works reported in the literature agree that Mn^3+^ and Mn^4+^ ions occupy preferably octahedral sites and Mn^2+^ is found in tetrahedral sites [41,42]. Based on the quantified results of the XPS analysis, which are summarised in Table 1, it is observed that Mn^3+^ dominates against Mn^2+^ and Mn^4+^ in the CuMnO_x_ film, i.e., Mn^3+^ > Mn^4+^ ≈ Mn^2+^. Thus, the results obtained in the XPS analysis support the XRD study confirming the formation of spinel-like materials in the first constituent absorber layer, concretely Cu_1_._5_Mn_1_._5_O_4_ and CuMn_2_O_4_ phases.
- CuFeMnO_x_
  Regarding the CuFeMnO_x_ constituent absorber film, Figure 3 shows the XPS spectra of Cu 2p (a), Mn 2p (b), Fe 2p (c) and the corresponding fit curves of Cu 2p_3/2_ (d), Mn 2p_3/2_ (e) and Fe 2p_3/2_ (f) peaks recorded for the film annealed at 600 °C. Concerning the Cu element, by performing peak-fitting deconvolution, the Cu 2p_3/2_ spectrum can be separated into two peaks: 932.7 and 934.8 eV, which correspond to the Cu^2+^ situated in octahedral and tetrahedral sites, respectively [38]. Apart from the information given by the peaks at the definite binding energies, the satellite peak at 938–945 eV in the XPS spectrum of Cu 2p (Figure 3a) also confirms the presence of Cu^2+^ in the layer under study. Therefore, it can be confirmed that only one oxidation state of copper (Cu^2+^) is detected in the CuFeMnO_x_ layer, preferably, occupying octahedral sites (85%) than tetrahedral sites (15%) as the quantified results of the XPS analysis corroborate.
  In relation to the manganese ion contribution, a co-existence of various oxidation states is expected for this ion given the complexity of the registered peaks. In fact, in Figure 3e it can be seen that the deconvolution curve of Mn 2p_3/2_ is composed of two peaks, namely, 640.8 and 642.7, corresponding to Mn^2+^ and Mn^4+^, respectively, and multiplet-split components associated with Mn oxides at binding energies higher than 644 eV. As mentioned in previous paragraphs and in accordance with the majority of the references, the Mn^2+^ is preferably found seizing tetrahedral sites whereas the Mn^4+^ has a tendency to occupy octahedral sites. Based on these results, no differences in terms of the oxidation states of manganese are observed between both CuMnO_x_ and CuFeMnO_x_ films. However, by analysing and comparing the quantified results obtained for the manganese species present in each CuMnO_x_ and CuFeMnO_x_ film, clear differences are noticed. Specifically, it is observed that Mn^2+^ dominates against Mn^4+^, i.e., Mn^2+^ > Mn^4+^ and no Mn^3+^ species are present on the CuFeMnO_x_ film, meanwhile in the CuMnO_x_ film the predominant manganese species is Mn^3+^ (Mn^3+^ > Mn^4+^ ≈ Mn^2+^).
  Regarding the iron cation, Figure 3c,f show the Fe 2p XPS spectrum as well as the fit curve of Fe 2p_3/2_. Since the position of the peaks Fe 2p_1/2_ and Fe 2p_3/2_ as well as their respective satellite positions depend closely on the ionic states of iron [43], from the experimental results it can be established that the CuFeMnO_x_ film is composed of a co-existence of Fe^2+^ and Fe^3+^. The presence of the main peak at the binding energy of 710.5 eV and the satellite peak at about 718.5 eV in the Fe 2p XPS spectrum are indicative of the presence of Fe^3+^ ions [11,44]. By performing a peak-fitting deconvolution of the Fe 2p_3/2_ spectrum, three different peaks at 710.5, 709.1 and 712.0 eV can be resolved. The peak situated around 709.1 eV confirms the presence of Fe^2+^ species meanwhile the peaks located at the binding energies of 710.5 and 712.0 indicate the presence of Fe^3+^ species in different coordination environments. Clearly, these results support the ones obtained in the XRD analysis in which two phases have been detected: FeMnCuO_4_ and Fe_3_O_4_. Regarding the site distribution of the iron ion, the majority of the references agree that Fe^3+^ ions occupy preferably octahedral sites (710.5 eV) in spinel structures rather than tetrahedral sites (712.0 eV) [44,45]. This statement is corroborated by analysing the quantified results obtained in Figure 3f, that is, 50% for Fe^3+^ ions occupying octahedral sites while 24% for Fe^3+^ ions occupying tetrahedral sites. Certainly, both XRD and XPS results support the formation of the spinel-like material of the FeMnCuO_4_ phase, as well as the presence of the segregated oxide of iron Fe_3_O_4_ in the second constituent absorber layer.

The data related to the oxidation-state percentage and site distribution of copper, manganese and iron ions in CuFeMnO_x_ thin film are summarised in Table 2.

**Figure 4:**
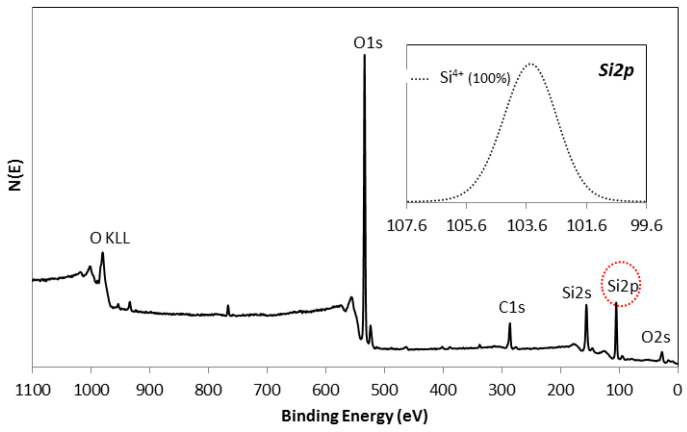
XPS survey spectrum of SiO_2_ coating sintered at 500 °C in the blower and in the inset the XPS spectra corresponding to the Si 2p.

- SiO_2_
  Since the XRD analysis of the SiO_2_ layer reports the formation of an amorphous phase of the silica film at the studied sintering conditions, the composition of this third layer was studied by using the XPS technique. Figure 4 shows the XPS survey spectrum for the silica layer sintered in the blower and the corresponding Si 2p spectrum (inset). Based on this graphical representation, it can be seen that the protective and antireflective SiO_2_ film is composed of Si and O. The presence of C in the spectrum is associated with the environmental contamination of the sample. According to these results, it can be noticed that the entire silicon ion in the layer is present as Si^4+^, being, therefore, part of the SiO_2_ layer in its entirety.

### 3.2. Effect of the SiO_2_ Sintering Process on the Optical Behaviour

**Figure 5:**
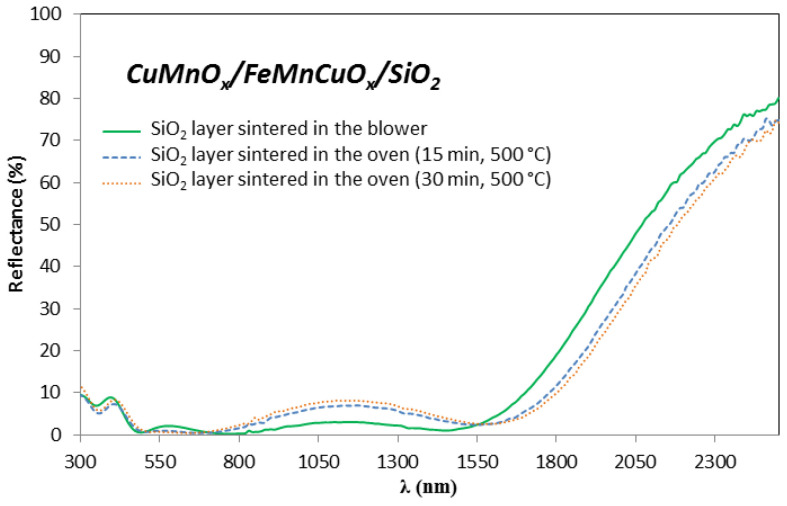
Influence of the sintering process of SiO_2_ coating on the hemispherical reflectance spectra of the absorber Al/CuMnOx/CuFeMnOx/SiO_2_.

In this section, a detailed study of the influence of the SiO_2_ sintering process on the optical parameters of the proposed absorber Al/CuMnO_x_/CuFeMnO_x_/SiO_2_ is presented. The SiO_2_ sintering was performed by using either a blower for 5 min or an oven for 15 or 30 min. In Figure 5 it can be seen how the variation of this SiO_2_ sintering process influences the hemispherical reflectance spectra of the complete absorber samples. Basically, higher values of reflectance in the second maximum (from 0.70 µm to the absorption edge) are reached for the absorber samples whose third layer was sintered in the oven during both 15 and 30 min (7–8% at 1.15 µm) instead of using the blower (3% at 1.15 µm). In addition, the absorbers whose SiO_2_ layer was sintered in the oven had the absorption edge shifted to higher wavelengths and showed a decrease in reflectance in the NIR range. Specifically, the absorption edge appears at around 1.60 µm for both samples whose silica layer was sintered in the oven while for the sample whose SiO_2_ layer was sintered in the blower the absorption edge is placed at 1.45 µm. Moreover, it was observed that as the sintering time in the oven increased, the second maximum reached slightly higher values of reflectance accompanied by a decrease in the reflectance in the NIR range.

**Figure 6:**
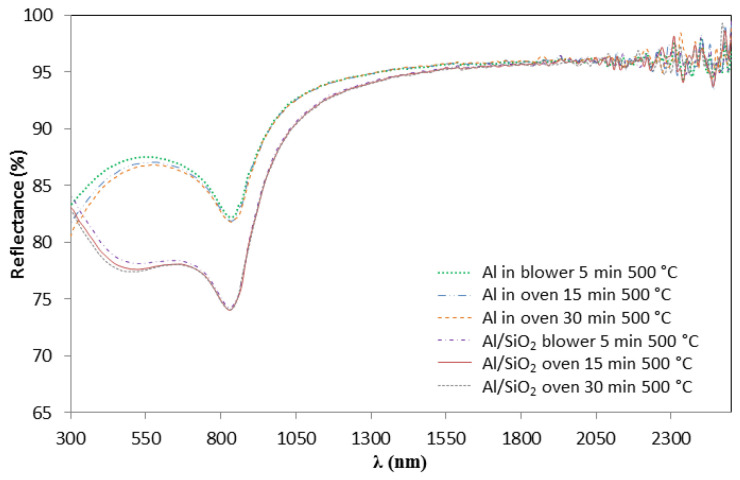
Effect of the SiO_2_ sintering conditions on the bare Al substrate and Al/SiO_2_ samples on the hemispherical reflectance measurements.

These variations in the curve shape of the Al/CuMnO_x_/CuFeMnO_x_/SiO_2_ samples, strongly suggest that thicker layers are obtained in case of sintering the SiO_2_ layer in the oven instead of sintering in the blower. This thicker layer resulting from the sintering process in the oven could be attributed to oxide layer formation between the aluminium substrate and the absorber as a consequence of the oxidation of the aluminium substrate at longer sintering times. This fact is confirmed in Figure 6, which shows the reflectance spectra of the bare aluminium substrate and of the SiO_2_ coated aluminium substrate (Al/SiO_2_) both treated under the same conditions. In the bare aluminium, the reflectance decreases with the increase in the sintering time (300–800 nm) suggesting the alumina layer formation. When the SiO_2_ is deposited, the same effect can be observed.

All the curve shape variations described in both Figure 5 and Figure 6 implied, as expected, changes in the absorptance values of the samples. As is shown in Table 3, a significant loss of the solar absorptance value can be found by sintering the silica layer in the oven (0.950–0.953) compared to using the blower (0.957).

### 3.3. Effect of the SiO_2_ Sintering Process on Durability

Apart from good optical properties, which have already been discussed in previous work for the proposed three-layer configuration absorber Al/CuMnO_x_/CuFeMnO_x_/SiO_2_, absorber coatings have to accomplish long-term stability during service life as the micro-climatic conditions can significantly affect the performance of the material. The effect of the SiO_2_ sintering conditions on durability has been also studied since this layer is often used as a protective layer as well as an anti-reflective [46]. Thermal stability tests and condensation tests were carried out separately for each sample.

**Figure 7:**
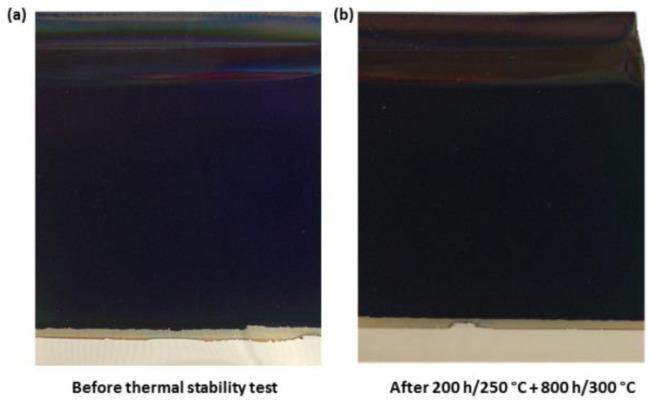
Photographs of the Al/CuMnO_x_/CuFeMnO_x_/SiO_2_ (blower) selective absorber before (**a**) and after (**b**) the thermal stability test.

**Figure 8:**
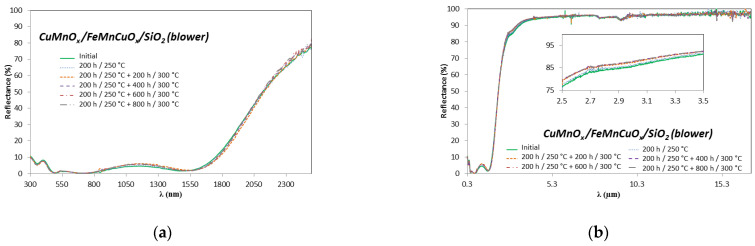
Hemispherical reflectance spectra of Al/CuMnO_x_/CuFeMnO_x_/SiO_2_ (blower) samples, before and after each ageing stage of the thermal stability test (**a**) in the solar wavelength range; (**b**) from 0.3 to 17 µm. Amplification between 2.5 and 3.5 µm (inset).

Regarding the thermal stability test, six replicas of each three-layer configuration sample whose SiO_2_ layer was sintered in the blower (5 min) or in the oven (15 min), were heated at 250 °C for 200 h and afterwards at 300 °C for 200 h, 400 h, 600 h and 800 h. After each degradation time interval, the samples were first visually inspected and no apparent deteriorations were observed. All the samples kept their initial appearances and the colour remained unchanged. As an example, photographs of the Al/CuMnO_x_/CuFeMnO_x_/SiO_2_ (blower) selective absorber before (a) and after (b) the thermal stability study are shown in Figure 7. Figure 8 displays the hemispherical reflectance spectra for the sample Al/CuMnO_x_/CuFeMnO_x_/SiO_2_ whose silica layer was sintered in the blower, before and after each ageing stage for the wavelength range 0.3–2.5 μm (Figure 8a) and 0.3–17 μm (Figure 8b). Both graphical representations show hardly any difference between the spectra, corroborating an almost non-existent modification of the silica films during the thermal stability test. It should be emphasised that this effect was also observed for the rest of the samples whose SiO_2_ layer was sintered in the oven for 15 min.

Seeing minimal variations in the hemispherical reflectance spectra, neither notable fluctuations in both solar absorptance and thermal emittance values were expected for the tested samples. Indeed, Table 4 summarises the negligible variations observed in the optical parameters of the samples before and after each exposure period as well as the resulting PC value.

Since the influence of ageing on the efficiency of the collector must be less than 5% after 25 years, the samples have to reach PC values lower than 0.05 at the end of the test to be accepted, as the standard specifies [29]. Values in Table 4 show that the optical performance of all the absorbers studied is reduced less than 5% of its original value during the design service life time period. In fact, each sample demonstrated to be very stable, with the PC values registered in all cases close to zero. As a result of these very small PC values, it can be considered that all the studied absorber surfaces are qualified as far as thermal stability is concerned, even after approximately 1000 h of testing, regardless of the sintering process of the SiO_2_ layer. Therefore, the excellent thermal stability results obtained indicate that the constituent absorber coatings are suitable for solar absorbers in terms of thermal stability performance. 

Following the long term durability study of the absorbers with different SiO_2_ sintering processes, a condensation test was performed. As a general trend, condensation tests are considered much more aggressive than thermal stability tests. This condensation test involved the exposure of six replicas of each tested absorber under constant condensation (40 °C) in the weathering chamber with interruptions to measure the extent of degradation after 150 h, 300 h and 600 h. At each interruption, the samples were first examined by visual inspection for evaluating the absorber coatings and then the optical measurements were performed to calculate the solar absorptance, thermal emittance and PC values.

**Figure 9:**
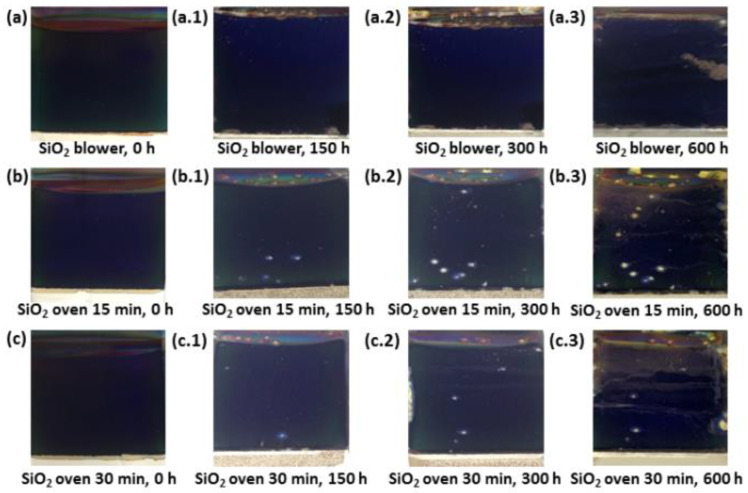
Photographs of Al/CuMnO_x_/CuFeMnO_x_/SiO_2_ samples with different sintering process of the silica layer before condensation test (**a**–**c**), after 150 h condensation exposure (**a.1**,**b.1**,**c.1**), after 300 h condensation exposure (**a.2**,**b.2**,**c.2**) and after 600 h condensation exposure (**a.3**,**b.3**,**c.3**).

Due to the combined effect of condensation, high humidity and temperature (40 °C), some colour changes and sample degradation were observed for the three types of aluminium based solar selective absorbers studied, as shown in Figure 9. Particularly, pitting corrosion and a minor colour degradation after the first 150–300 h of testing were observed for the samples analysed.

**Figure 10:**
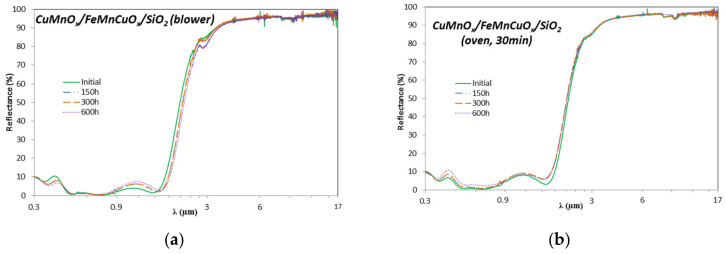
Variation of the hemispherical reflectance spectra for wavelengths from 0.3 to 17 µm of Al/CuMnO_x_/CuFeMnO_x_/SiO_2_ (blower) (**a**) and Al/CuMnO_x_/CuFeMnO_x_/SiO_2_ (oven, 30 min) (**b**) samples before and after each ageing step in the condensation test.

After each testing time in the weathering chamber, the reflectance spectra of the samples were also measured and the absorbers’ optical performance was determined. Figure 10 shows the variations registered in the hemispherical reflectance spectra of Al/CuMnO_x_/CuFeMnO_x_/SiO_2_ (blower) and Al/CuMnO_x_/CuFeMnO_x_/SiO_2_ (oven 30 min) samples before and after each ageing step in the weathering chamber. Both representations are selected in this manuscript due to the interesting conclusions reached in this study, being clearly visible the variations produced in the spectra during the test. The variations observed occur mainly in the solar wavelength range of 0.3–2.5 µm. In the specific case of the Al/CuMnO_x_/CuFeMnO_x_/SiO_2_ (blower) sample (Figure 10a)), it is observed that the first maximum (around 0.4 µm) reaches lower values of reflectance (10.0–6.8%) as the condensation test progresses. In addition, an increase of the second maximum (0.80 µm until the absorption edge), a displacement of the absorption edge to higher wavelengths and a decrease in the NIR range are also observed. These variations as the test time proceeds are characteristic of an increase in the layer thickness [16]. However, as this effect is improbable in these conditions, the variations observed in the curve shape of the Al/CuMnO_x_/CuFeMnO_x_/SiO_2_ (blower) samples after the condensation test could be attributed to the oxidation of the aluminium substrate. On the other hand, a different tendency is observed in the Al/CuMnO_x_/CuFeMnO_x_/SiO_2_ (oven 30 min) samples (Figure 10b)). In this case, the first maximum (around 0.4 µm) of the sample spectrum reaches higher values of reflectance as the condensation test progresses (7.5–11%). Meanwhile the second maximum (0.80 µm until the absorption edge) maintains nearly its original position around the reflectance value of 8.5%. Apart from these variations, it is also observed a slight shift of the absorption edge to lower wavelength values as the test progresses. Definitely, these variations in the shape of the Al/CuMnO_x_/CuFeMnO_x_/SiO_2_ (oven 30 min) samples spectra suggest that the silica antireflective layer becomes thinner as the test in the weathering chamber progresses, that is, the SiO_2_ over-coating degrades along the condensation test. 

Optical parameters as well as the resulting PC values registered for each sample before and after each cycle of the condensation test are recorded in Table 5. According to optical parameters analysis, a slight decrease of the solar absorptance value and an increase of the thermal emittance value with the exposure were observed for all the Al/CuMnO_x_/CuFeMnO_x_/SiO_2_ samples studied. There is an exception for exposure times between 150 and 300 h in which the values of solar absorptance, thermal emittance and PC remained unaltered. 

In spite of the changes observed in the optical properties values and aspects of the samples after the condensation test, it can be concluded that all the absorbers studied registered a final PC value well below the critical value for which an absorber can be considered qualified, that is, the absorbers’ optical performance was still good for all samples with a PC ≤ 0.015 (Table 5) after 600 h of testing at 40 °C. 

**Figure 11:**
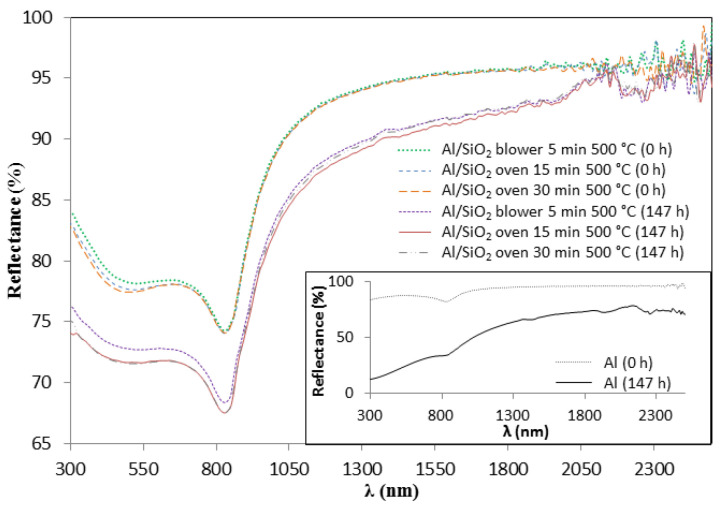
Variation of the hemispherical reflectance spectra of the Al/SiO_2_ samples before and after 147 h of condensation test. Hemispherical reflectance spectra of the bare Al substrate after 147 h of condensation test (inset).

**Figure 12:**
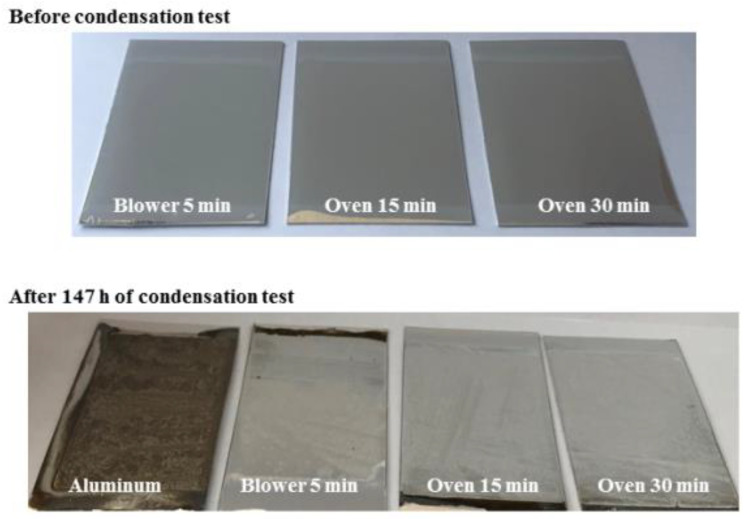
Photographs of bare Al and Al/SiO_2_ samples before and after 147 h of condensation test.

Additional comparative experiments were carried out in order to delve into the role that the alumina layer produced during the preparation of the three-layer complete absorber and the silica top layer plays in protecting the samples against constant condensation conditions. For this purpose, preheated Al at 600 °C (simulating the sintering treatments of the complete absorber) and not-preheated were used to apply the upper silica layer and were tested in the condensation chamber (three replicas). Figure 11 shows the hemispherical reflectance spectra of not-preheated Al/SiO_2_ samples before and after 147 h of condensation test. The reflectance decreases considerably (around 8%) for all the samples and these variations demonstrate the degradation that the three samples undergo at the condensation conditions tested. The inset in Figure 11 also shows the variation of the hemispherical reflectance of bare aluminium before and after 147 h of condensation test. It should be noted that there is a drastic reflectance decrease, from 89% to 37% after this testing time. Figure 12 shows the photos of the samples before and after 147 h of testing. It can be observed that the SiO_2_ layer protects to a great extent the Al substrate from oxidation but not enough to avoid being unaltered. The Al/SiO_2_ samples exhibited clear degradation, going from being transparent and specular before the condensation test to being whitish and matte after 147 h of testing. On the other hand, the bare Al appeared completely oxidised and degraded in the same conditions.

**Figure 13:**
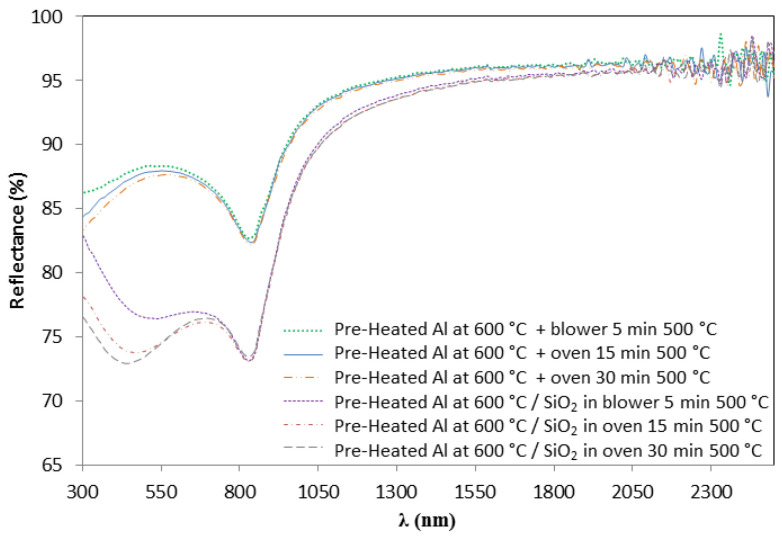
Effect of SiO_2_ sintering conditions on both preheated bare Al and Al/SiO_2_ on the hemispherical reflectance spectra.

The hemispherical reflectance spectra of Al/SiO_2_ preheated before applying the silica layer together with bare aluminium preheated and heated (simulating the sintering treatment of the SiO_2_ layer) are plotted in Figure 13. The preheating treatment of the aluminium substrate induces greater variations in the reflectance spectra between the different SiO_2_ sintering conditions. Furthermore, better results regarding durability in the climate chamber were obtained in comparison with the non-pre-heated Al samples. In fact, the pre-heating of Al/SiO_2_ samples at 600 °C allowed all the analysed samples to remain intact after 600 h of condensation testing regardless of the SiO_2_ sintering process. The solar reflectance data after each degradation interval during the condensation test of the preheated Al/SiO_2_ samples are reported in Table 6. All the replicas tested duplicate the pristine hemispherical reflectance spectra. These results confirm that the good resistance to the humidity of the proposed absorber is due to the protective action of both the silica layer and alumina layer produced naturally during the sintering process of the whole absorber preparation. The antireflective SiO_2_ layer protects, to some extent, the aluminium from oxidation but is not enough to withstand the condensation test conditions. 

Even though the three kinds of absorbers studied can be qualified with respect to their resistance to high temperatures (stability test) and constant condensation conditions (condensation test), it would be interesting to analyse how the sintering conditions of the silica can affect the wetting ability of the absorber surface. For this reason, static contact angle measurements were carried out before subjecting the samples to any test and the results are recorded in Table 7. It can be observed from the measurements that the different silica sintering processes studied affect differently the wetting ability of the absorber surface. In particular, the static contact angle value decreases from 37° to 25–21° depending on whether the SiO_2_ anti-reflective coating has been sintered in the blower or oven. Specifically, the highest static contact angle value was obtained for the absorber whose silica layer was sintered in the blower while the lowest static contact angle value was registered for the absorber whose SiO_2_ layer was sintered in the oven for 30 min. These results support that the longer the SiO_2_ layer is sintered in the oven, the more organic matter is burnt making the layer more hydrophilic and then the moisture of the condensation test stays longer in the sample as the surface becomes wetter.

In conclusion, based on the results obtained in both degradation tests of thermal stability and condensation resistance (absorptance values, thermal emittance and PC ≤ 0.015), it can be confirmed that the CuMnO_x_/CuFeMnO_x_ films were effectively protected by the combined action of the alumina layer produced onto the aluminium substrate during the absorber preparation and the SiO_2_ over-coating; regardless of the sintering process used for the SiO_2_ layer. In this way, it can be summarised that optimal results were obtained for any of the samples studied.

**Figure 14:**
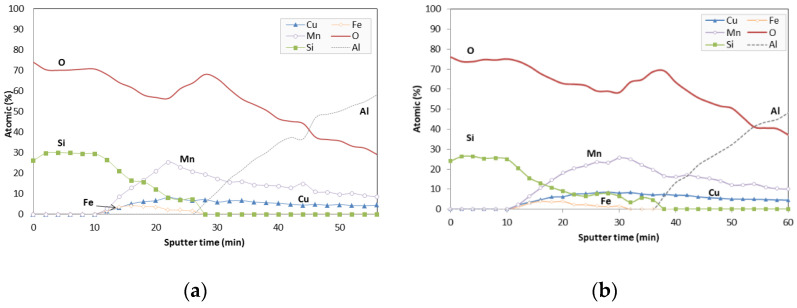
XPS depth profile of the three-layered selective absorber before (**a**) and after 600 h at 40 °C (**b**). Variation of the element concentrations as a function of Ar+ ion etch time.

Once both durability and characterisation studies have been completed, a depth profile study of the selective absorber Al-CuMnO_x_/CuFeMnO_x_/SiO_2_ whose silica layer was sintered in the blower was carried out by using XPS. This three layer-configuration sample was successfully etched with an Ar^+^ ion beam. Figure 14 shows the results of the depth profile study of this configuration before (a) and after (b) the ageing tests. It can be seen that the silicon concentration decreases gradually as the etching proceeds whereas the copper, manganese and iron concentrations firstly increase, attain saturation and finally decrease gradually with etching time. Immediately after the etching reaches the Al substrate, an increase in the concentration of the Al, together with an increase in the oxygen content, are observed in both pristine and aged samples. This fact confirms the formation of the alumina layer onto the aluminium substrate during the absorber sintering. Additionally, in aged samples, (Figure 14b), a hydration phenomenon of spinel components was perceived as a consequence of the constant humidity conditions to which the samples have been subjected. The iron spinel stops appearing 28 min after sputtering in the case of the pristine samples while in the case of the tested samples the iron stops appearing after approximately 40 min of sputtering.

The XPS depth profile results demonstrate the formation of the protective alumina layer during the sintering of the spinel layers and, on the other hand, they clearly suggest that the variations produced in the optical properties of samples tested under condensation could be produced by a hydration process of the CuFeMnO_x_ intermediate films.

## 4. Conclusions

In this paper, the structural characterisation of the constituent absorber films was studied. The XRD and XPS results, confirm the formation of spinel-like materials in the first absorber layer with two different stequiometries, Cu_1_._5_Mn_1_._5_O_4_ being the main phase. With regards to the second layer, the co-existence of the spinel CuFeMnO_4_ with a secondary magnetite phase of Fe_3_O_4_ has been demonstrated. Additionally, durability tests of this competitive three-layer configuration solar selective absorber prepared by dip-coating have been presented. The results obtained showed that the sintering conditions of the SiO_2_ top layer affect the optical properties as well as the accelerated ageing test results. In general, all the sintering conditions used gave place to selective absorbers with very good optical performance (α_s_ ≈ 0.957 and ε_t_ (100 °C) ≈ 0.038) and excellent durability results. Specifically, PC values close to 0.001 in the thermal stability test and below 0.015 in the condensation test were obtained. On the other side, it was shown that the protective action against condensation results from the combination of both alumina layers produced during the absorber layer preparation and the antireflective silica layer. A hydration phenomenon of Fe spinel is confirmed by XPS for tested samples as a consequence of the constant humidity conditions to which the samples are subjected during the climatic chamber test. The results obtained in this work validate the proposed structure Al/CuMnO_x_/CuFeMnO_x_/SiO_2_ as a very promising selective absorber for low temperature solar thermal applications.

## Figures and Tables

**Table 1:** Oxidation state and site distribution of copper and manganese ions in CuMnO_x_ thin film. The percentages of Mn species were recalculated once the multiplet split components contributions (higher than 644 eV) were removed.

Compound	Cu 2p_3/2_				Mn 2p_3/2_		
CuMnO_x_	Cu^2+^ _Td_ 935.5 eV (%)	Cu^2+^ _Oh_ 933.3 eV (%)	Cu^0^ 931.8 eV (%)	Cu^+^ _Td_ 930.0 eV (%)	Mn^4+^_Oh_ 642.9 eV (%)	Mn^3+^ _Oh_ 641.5 eV (%)	Mn^2+^ _Td_ 640.0 eV (%)
	4	44.3	23.9	27.8	21.2	48.1	30.7

**Table 2:** Oxidation state and site distribution of iron, manganese and copper ions in FeMnCuO_x_ thin film.

Compound	Cu 2p_3/2_		Fe 2p_3/2_			Mn 2p_3/2_	
CuFeMnO_x_	Cu^2+^ _Td_ 934.8 eV (%)	Cu^2+^ _Oh_ 932.7 eV (%)	Fe^3+^ _Td_ 712.0 eV (%)	Fe^3+^ _Oh_ 710.5 eV (%)	Fe^2+^ 709.2 eV (%)	Mn^4+^_Oh_ 642.7 eV (%)	Mn^2+^ _Td_ 640.8 eV (%)
	18.7	81.3	19.1	54.6	26.3	24.9	75.1

**Table 3:** Solar absorptance values of Al/CuMnO_x_/CuFeMnO_x_/SiO_2_ samples with different sintering processes of the silica layer.

Sintering Process of SiO_2_ Antireflective Coating	Solar Absorptance (α_s_)
Blower	0.957
Oven, 15 min 500 °C	0.953
Oven, 30 min 500 °C	0.950

**Table 4:** Solar absorptance, thermal emittance and PC values calculated before and after different degradation intervals for the samples during the thermal stability test. All the values are the average of the values obtained for the six replicas.

Samples/SiO_2_ Sintering Process	Solar Absorptance (α_s_)	Thermal Emittance (ε_t_, 100 °C)	PC
*CuMnO_x_/CuFeMnO_x_/SiO_2_ blower*			
Initial	0.957	0.038	
200 h/250 °C	0.957	0.038	0
200 h/250 °C + 200 h/300 °C	0.956	0.038	0.0012
200 h/250 °C + 400 h/300 °C	0.956	0.038	0.0014
200 h/250 °C + 600 h/300 °C	0.956	0.038	0.0014
200 h/250 °C + 800 h/300 °C	0.956	0.038	0.0014
*CuMnO_x_/CuFeMnO_x_/SiO_2_ oven, 15 min*			
Initial	0.956	0.038	
200 h/250 °C	0.956	0.033	0
200 h/250 °C + 200 h/300 °C	0.953	0.035	0.0014
200 h/250 °C + 400 h/300 °C	0.954	0.034	0.0001
200 h/250 °C + 600 h/300 °C	0.954	0.034	0.0001
200 h/250 °C + 800 h/300 °C	0.954	0.034	0.0001

**Table 5:** Solar absorptance, thermal emittance and PC values calculated before and after each degradation interval for the samples during the condensation test at 40 °C. All the values are the average of the values obtained for the six replicas.

Samples/SiO_2_ Sintering Process	Solar Absorptance (α_s_)	Thermal Emittance (ε_t_, 100 °C)	PC
*CuMnO_x_/CuFeMnO_x_/SiO_2_ blower*			
Initial	0.957	0.038	
150 h/40 °C	0.955	0.036	0.0010
300 h/40 °C	0.955	0.036	0.0010
600 h/40 °C	0.954	0.038	0.0030
*CuMnO_x_/CuFeMnO_x_/SiO_2_ oven, 15 min*			
Initial	0.953	0.035	
150 h/40 °C	0.950	0.039	0.0055
300 h/40 °C	0.950	0.039	0.0055
600 h/40 °C	0.945	0.042	0.0113
*CuMnO_x_/CuFeMnO_x_/SiO_2_ oven, 30 min*			
Initial	0.950	0.039	
150 h/40 °C	0.945	0.039	0.0051
300 h/40 °C	0.945	0.039	0.0051
600 h/40 °C	0.935	0.040	0.0145

**Table 6:** Solar reflectance data after each degradation interval during the condensation test at 40 °C. Data correspond to preheated Al/SiO_2_ samples at different sintering conditions.

Sintering Conditions	Solar Reflectance (ρ_s,h_)								
	Replica	0 h	25 h	48 h	72 h	144 h	306 h	450 h	606 h
Blower	A	0.814	0.812	0.812	0.812	0.812	0.811	0.811	0.810
	B	0.815	0.814	0.814	0.815	0.815	0.813	0.813	0.812
	C	0.815	0.814	0.814	0.814	0.814	0.813	0.813	0.812
	Average	0.815	0.813	0.813	0.813	0.813	0.812	0.812	0.811
Oven, 15 min	A	0.801	0.800	0.800	0.800	0.799	0.798	0.799	0.799
	B	0.801	0.802	0.800	0.800	0.800	0.799	0.797	0.797
	C	0.803	0.802	0.802	0.802	0.801	0.799	0.799	0.798
	Average	0.802	0.801	0.801	0.801	0.800	0.799	0.799	0.798
Oven, 30 min	A	0.800	0.797	0.797	0.797	0.797	0.796	0.796	0.795
	B	0.805	0.802	0.802	0.802	0.802	0.800	0.799	0.798
	C	0.802	0.799	0.799	0.800	0.798	0.797	0.797	0.796
	Average	0.802	0.799	0.799	0.799	0.799	0.798	0.797	0.797

**Table 7:** Static contact angle values of Al/CuMnO_x_/CuFeMnO_x_/SiO_2_ samples with different sintering processes of the silica layer. The values are an average of four measurements.

Sintering Process of SiO_2_ Antireflective Coating	Static Contact Angle (°)
Blower	37
Oven, 15 min 500 °C	25
Oven, 30 min 500 °C	21
